# Author Correction: Defining the relative and combined contribution of CTCF and CTCFL to genomic regulation

**DOI:** 10.1186/s13059-020-02056-6

**Published:** 2020-06-02

**Authors:** Mayilaadumveettil Nishana, Caryn Ha, Javier Rodriguez-Hernaez, Ali Ranjbaran, Erica Chio, Elphege P. Nora, Sana B. Badri, Andreas Kloetgen, Benoit G. Bruneau, Aristotelis Tsirigos, Jane A. Skok

**Affiliations:** 1 Department of Pathology, New York University Langone Health, New York, NY 10016 USA; 2 Gladstone Institutes, San Francisco, CA 94158 USA; 3 Roddenberry Center for Stem Cell Biology and Medicine at Gladstone, San Francisco, CA 94158 USA; 4 Cardiovascular Research Institute, University of California, San Francisco, CA 94158 USA; 5 Applied Bioinformatics Laboratories, NYU School of Medicine, New York, NY 10016 USA; 6 Department of Pediatrics, University of California, San Francisco, CA 94158 USA; 7 Laura and Isaac Perlmutter Cancer Center, NYU School of Medicine, New York, NY 10016 USA

**Correction to: Genome Biol 21, 108 (2020)**

**https://doi.org/10.1186/s13059-020-02024-0**

Following publication of the original paper [1], the authors reported an error in the acknowledgements section. The updated acknowledgements section is given below and the changes have been highlighted in **bold typeface**.
