# Supplementary material for: Defining the relative and combined contribution of CTCF and CTCFL to genomic regulation
Source: Genome Biol. 2020 May 11;21:108. doi: 10.1186/s13059-020-02024-0 (PMC7212617; doi:10.1186/s13059-020-02024-0)
Supplement: Supplementary file 1 — Supplementary figure S1 to S7. [file 13059_2020_2024_MOESM1_ESM.pdf]

Fig S1

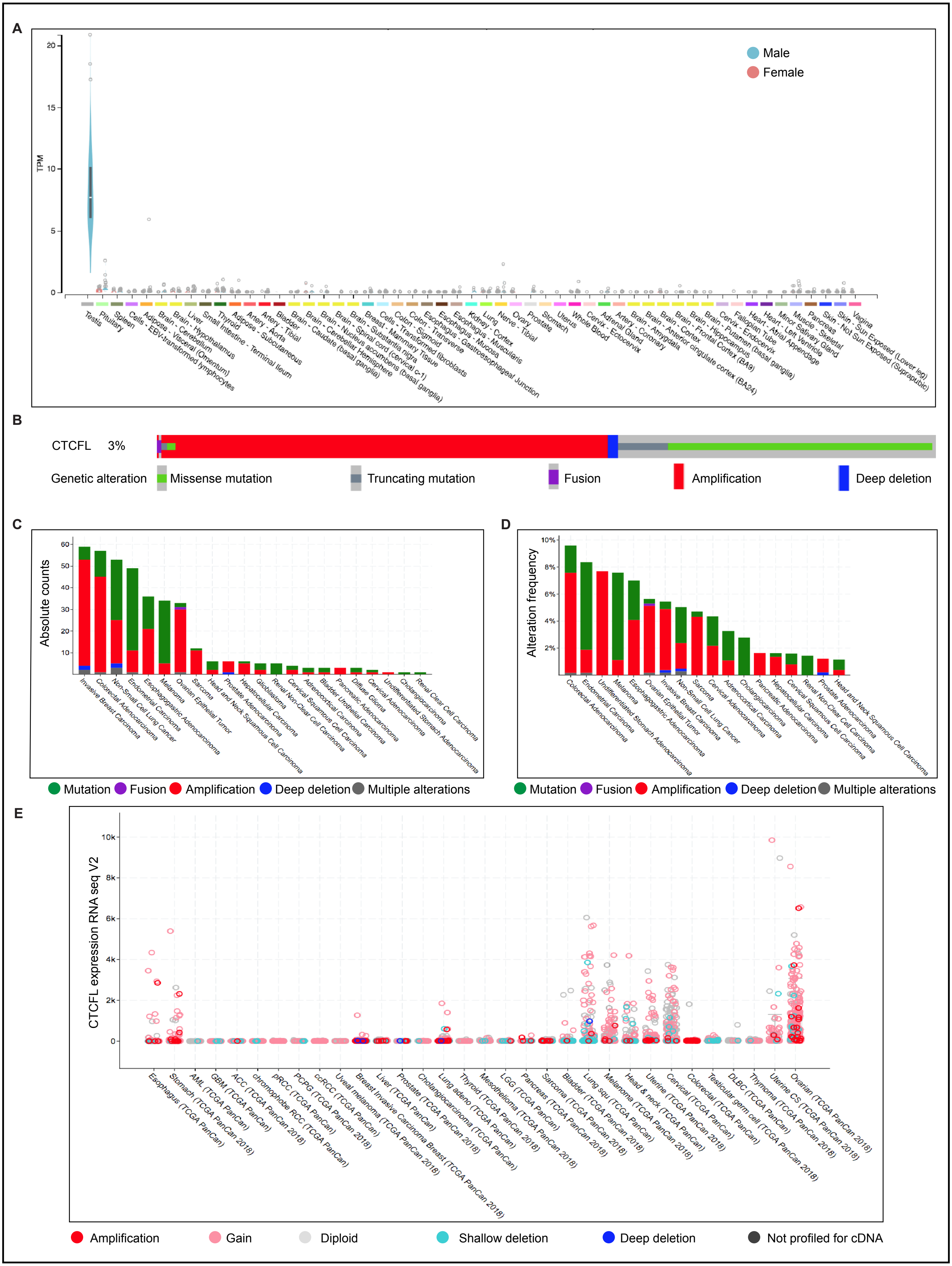

A
Male
Female
TPM
B
CTCFL 3%
Genetic alteration
Missense mutation
Truncating mutation
Fusion
Amplification
Deep deletion
C
Absolute counts
D
Alteration frequency
Mutation
Fusion
Amplification
Deep deletion
Multiple alterations
E
CTCFL expression RNA seq V2
Amplification
Gain
Diploid
Shallow deletion
Deep deletion
Not profiled for cDNA

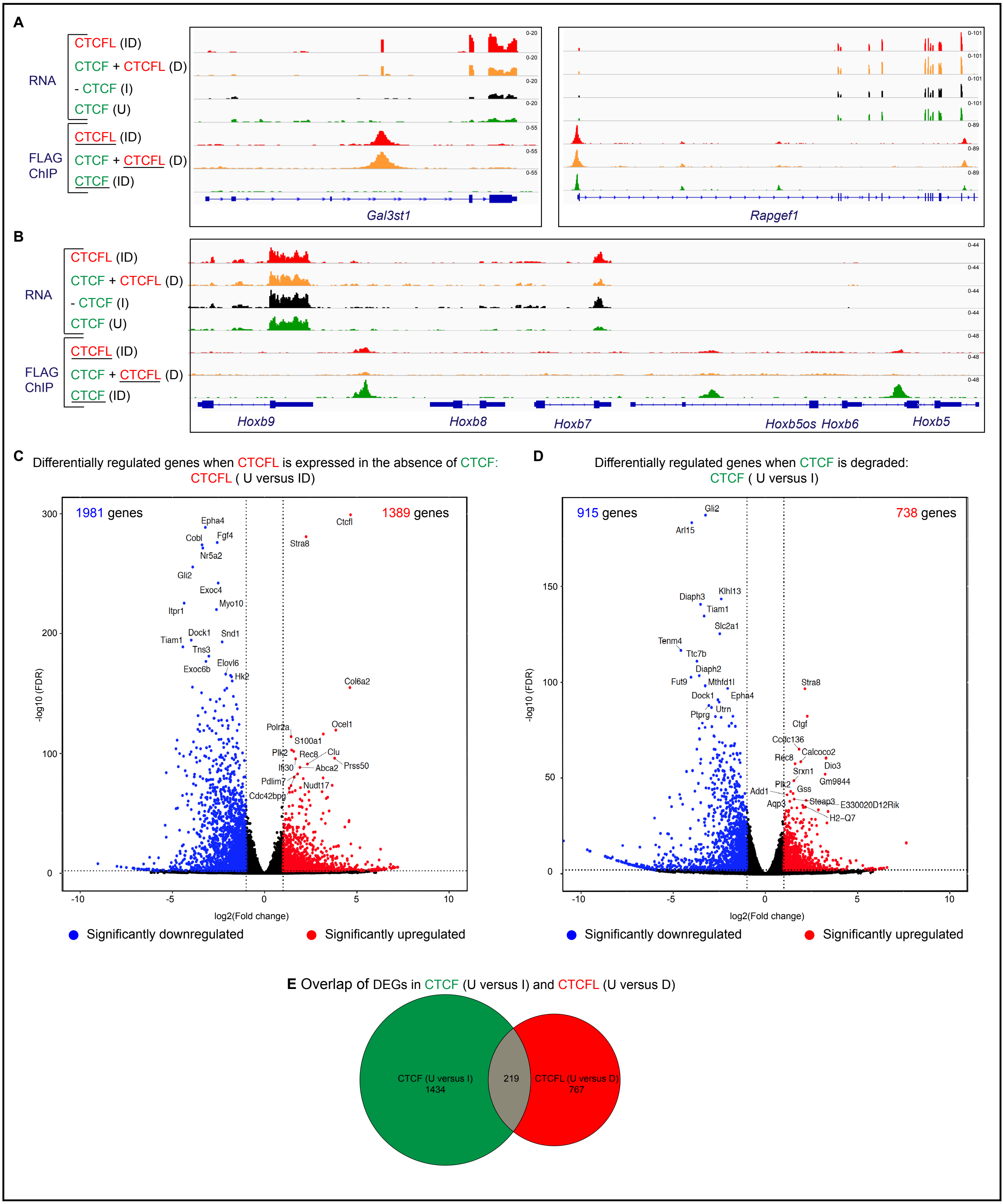
A
RNA
CTCFL (ID)
CTCF + CTCFL (D)
- CTCF (I)
CTCF (U)
FLAG ChIP
CTCFL (ID)
CTCF + CTCFL (D)
CTCF (ID)
Gal3st1
Rapgef1
B
Hoxb9
Hoxb8
Hoxb7
Hoxb5os Hoxb6
Hoxb5
C Differentially regulated genes when CTCFL is expressed in the absence of CTCF:
CTCFL (U versus ID)
1981 genes
1389 genes
-log10 (FDR)
log2(Fold change)
D Differentially regulated genes when CTCF is degraded:
CTCF (U versus I)
915 genes
738 genes
Significantly downregulated
Significantly upregulated
E Overlap of DEGs in CTCF (U versus I) and CTCFL (U versus D)
CTCF (U versus I)
1434
219
CTCFL (U versus D)
767

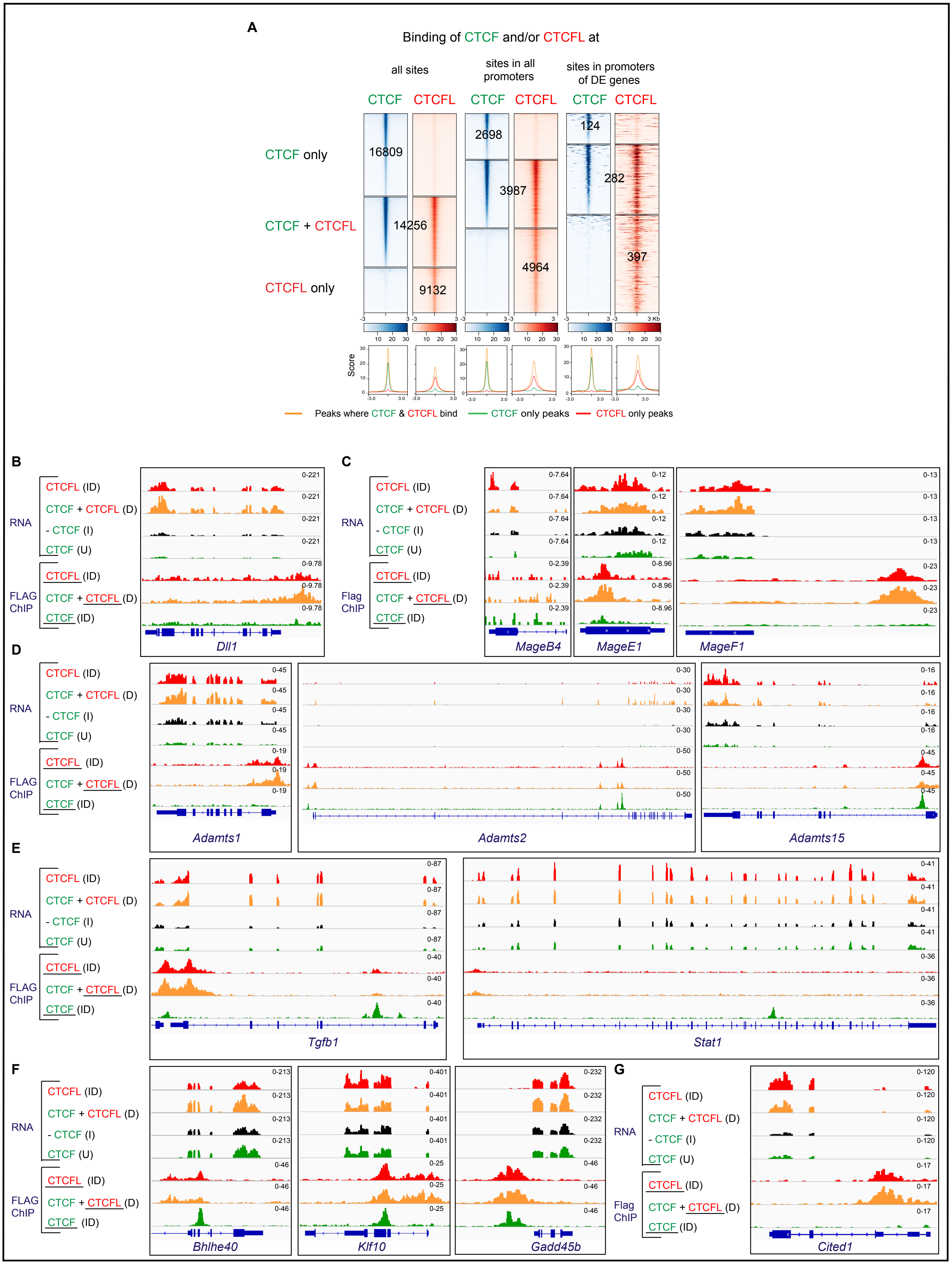
A
Binding of CTCF and/or CTCFL at
all sites
sites in all promoters
sites in promoters of DE genes
CTCF CTCFL CTCF CTCFL CTCF CTCFL
CTCF only
CTCF + CTCFL
CTCFL only
16809
14256
9132
2698
3987
4964
124
282
397
Score
Peaks where CTCF & CTCFL bind
CTCF only peaks
CTCFL only peaks
B
RNA
CTCFL (ID)
CTCF + CTCFL (D)
- CTCF (I)
CTCF (U)
FLAG ChIP
Dll1
C
Flag ChIP
MageB4
MageE1
MageF1
D
Adamts1
Adamts2
Adamts15
E
Tgfb1
Stat1
F
Bhlhe40
Klf10
Gadd45b
G
Cited1

Fig S4

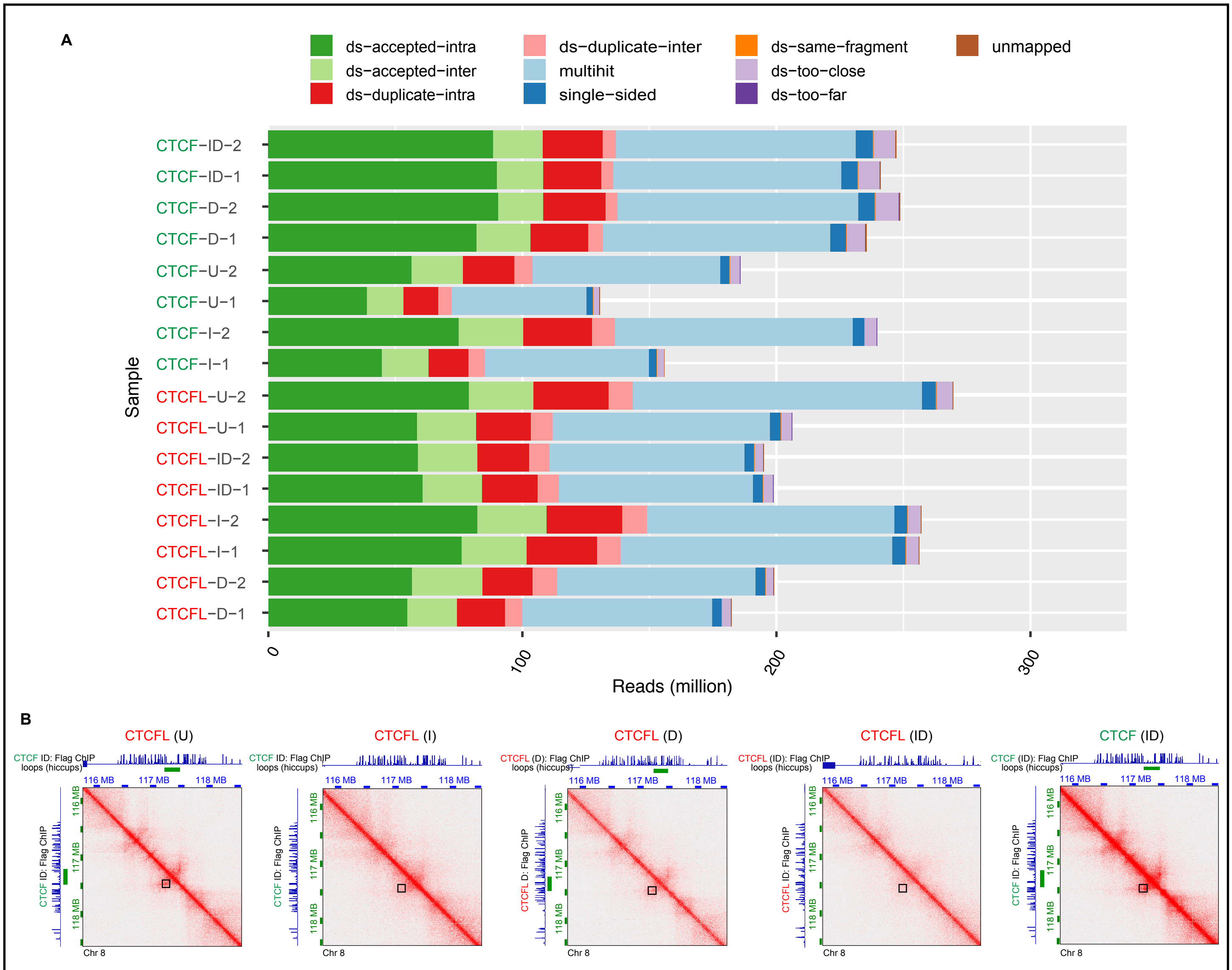
A
ds−accepted−intra
ds−accepted−inter
ds−duplicate−intra
ds−duplicate−inter
multihit
single−sided
ds−same−fragment
ds−too−close
ds−too−far
unmapped
Sample
CTCF−ID−2
CTCF−ID−1
CTCF−D−2
CTCF−D−1
CTCF−U−2
CTCF−U−1
CTCF−I−2
CTCF−I−1
CTCFL−U−2
CTCFL−U−1
CTCFL−ID−2
CTCFL−ID−1
CTCFL−I−2
CTCFL−I−1
CTCFL−D−2
CTCFL−D−1
0
100
200
300
Reads (million)
B
CTCFL (U)
CTCFL (I)
CTCFL (D)
CTCFL (ID)
CTCF (ID)
CTCF ID: Flag ChIP
loops (hiccups)
CTCFL (D): Flag ChIP
CTCFL (ID): Flag ChIP
CTCF (ID): Flag ChIP
116 MB
117 MB
118 MB
CTCFL D: Flag ChIP
CTCFL ID: Flag ChIP
Chr 8

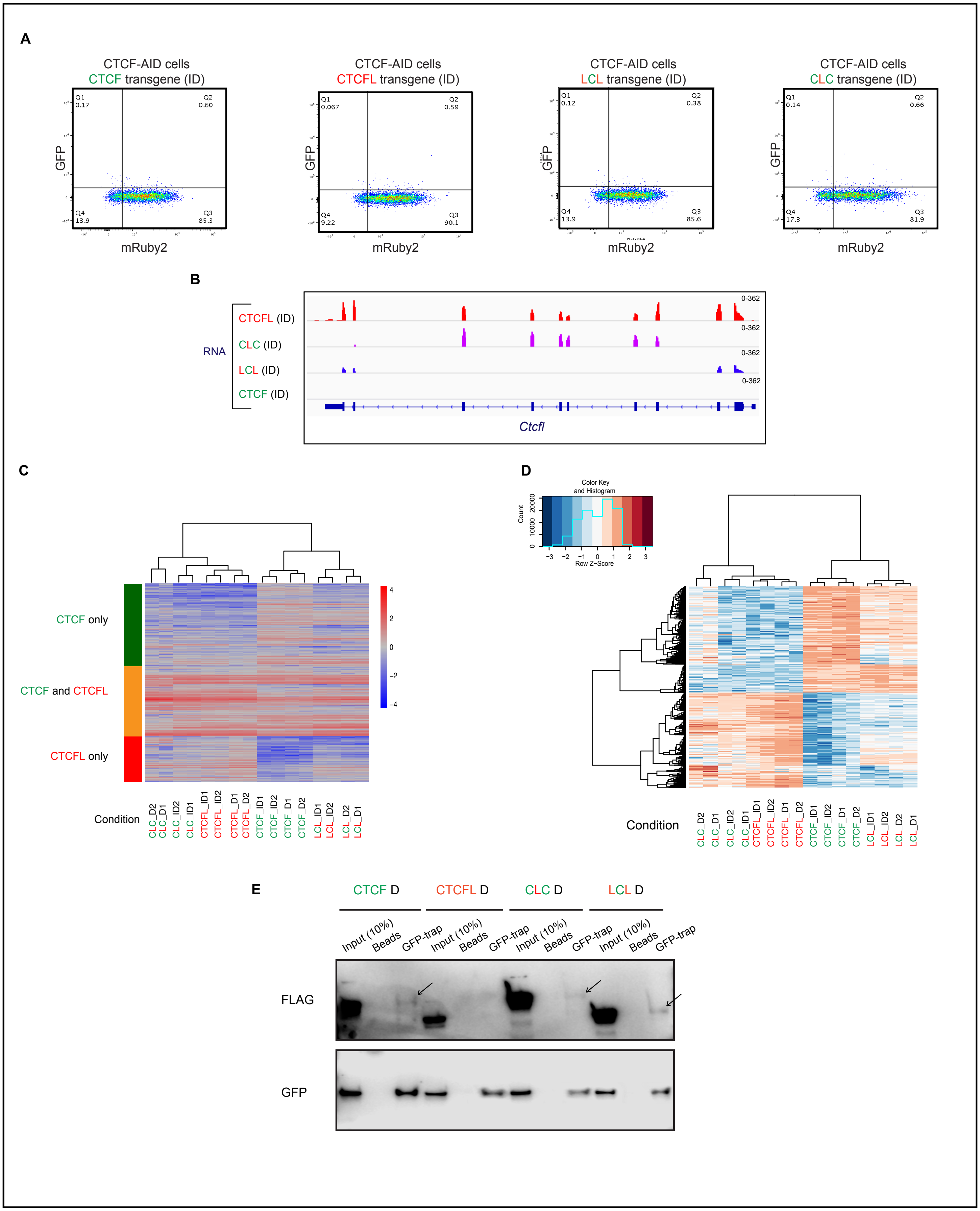
A
CTCF-AID cells
CTCF transgene (ID)
CTCFL transgene (ID)
LCL transgene (ID)
CLC transgene (ID)
GFP
mRuby2
Q1 0.17
Q2 0.60
Q4 13.9
Q3 85.3
Q1 0.067
Q2 0.59
Q4 9.22
Q3 90.1
Q1 0.12
Q2 0.38
Q4 13.9
Q3 85.6
Q1 0.14
Q2 0.66
Q4 17.3
Q3 81.9
B
RNA
CTCFL (ID)
CLC (ID)
LCL (ID)
CTCF (ID)
0-362
Ctcfl
C
CTCF only
CTCF and CTCFL
CTCFL only
Condition
CLC_D2
CLC_D1
CLC_ID2
CLC_ID1
CTCFL_ID1
CTCFL_ID2
CTCFL_D1
CTCFL_D2
CTCF_ID1
CTCF_ID2
CTCF_D1
CTCF_D2
LCL_ID1
LCL_ID2
LCL_D2
LCL_D1
D
Color Key and Histogram
Count
Row Z-Score
Condition
E
CTCF D
CTCFL D
CLC D
LCL D
Input (10%)
Beads
GFP-trap
FLAG
GFP

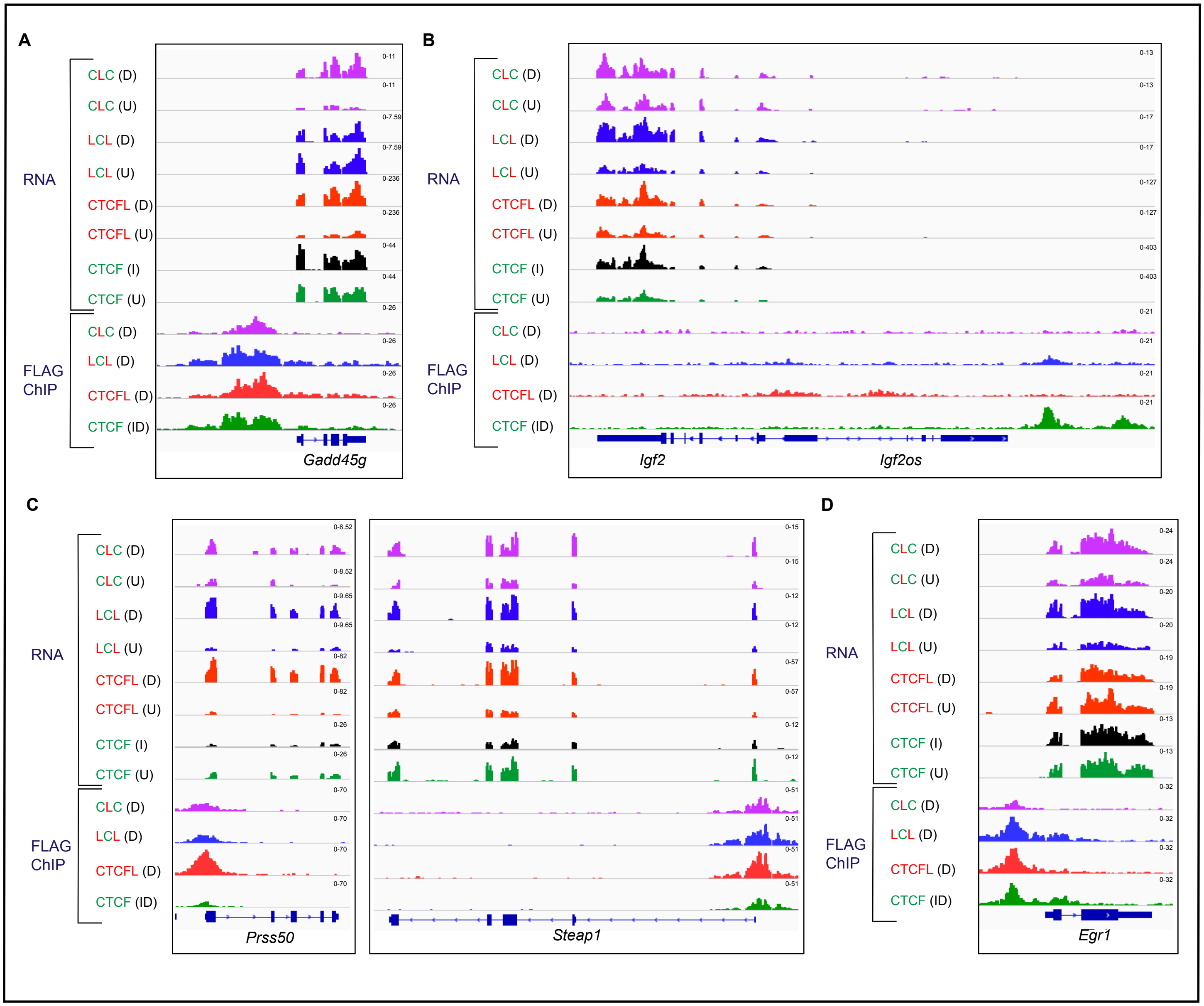
A
RNA
CLC (D)
CLC (U)
LCL (D)
LCL (U)
CTCFL (D)
CTCFL (U)
CTCF (I)
CTCF (U)
FLAG ChIP
CLC (D)
LCL (D)
CTCFL (D)
CTCF (ID)
Gadd45g
B
Igf2
Igf2os
C
Prss50
Steap1
D
Egr1

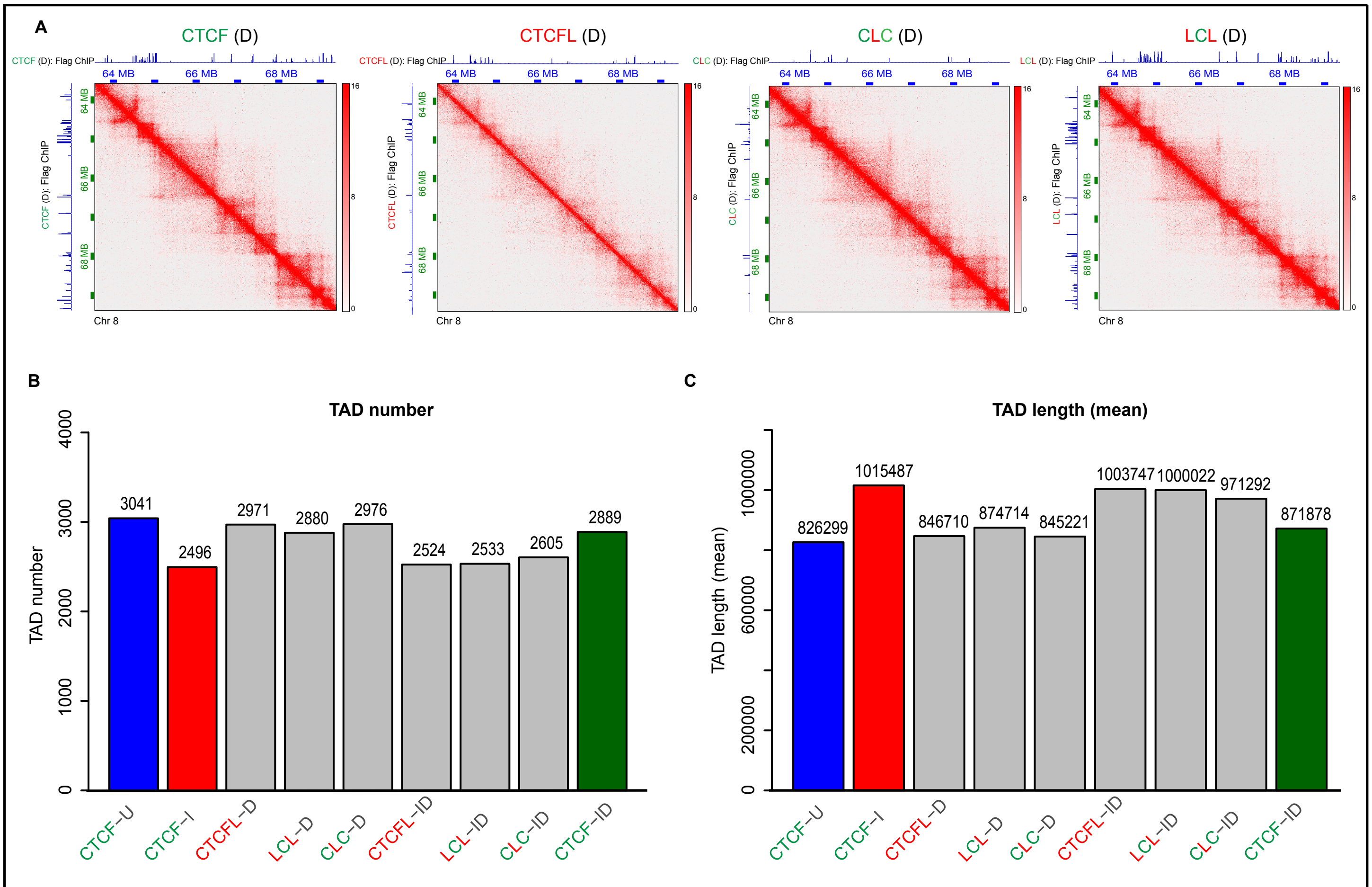
A
CTCF (D)
CTCFL (D)
CLC (D)
LCL (D)
CTCF (D): Flag ChIP
CTCFL (D): Flag ChIP
CLC (D): Flag ChIP
LCL (D): Flag ChIP
64 MB
66 MB
68 MB
Chr 8
16
8
0
B
TAD number
3041
2496
2971
2880
2976
2524
2533
2605
2889
CTCF–U
CTCF–I
CTCFL–D
LCL–D
CLC–D
CTCFL–ID
LCL–ID
CLC–ID
CTCF–ID
0
1000
2000
3000
4000
C
TAD length (mean)
826299
1015487
846710
874714
845221
1003747
1000022
971292
871878
0
200000
600000
1000000

Supplementary Figure Legends

Fig S1. CTCFL is genetically altered and aberrantly expressed in numerous cancers. A CTCFL is expressed only in testes in healthy individuals. Gene expression data for CTCFL (ENSG00000124092.8) was retrieved from GtexPortal (GTEx Analysis Release V7; dbGaP Accession phs000424.v7.p2). Data is sorted by median expression on a linear scale with males marked by blue and females by pink. **B** *CTCFL* is altered in 3% of cancer patients/samples profiled in cBioportal. Data was retrieved from Oncoprint of cBioPortal. The distribution of various alterations (missense mutation, truncations, fusion, amplification and deletion) in *CTCFL* in cancer are shown. As of 06/30/19, 3% of cancer patients profiled in cBioPortal had an alteration in *CTCFL*, the ratio of altered / profiled being 382 / 10950. **C, D** *CTCFL* is amplified across several cancer types. Alterations such as mutation, fusion, amplification and deletion reported in cancer patients are color coded. Absolute count of the patients (**C**) and alteration frequency in percentage (**D**) in various cancer types are shown. **E** CTCFL expression (RNA seq) and copy number variation in several cancer types. Data is sorted by median on a linear scale. Copy number alterations such as amplification, gain, diploid, deep and shallow deletion etc. are color coded.

Fig S2. CTCF and CTCFL bind to and regulate distinct sets of genes. A, B IgV tracks showing RNA-seq in all four conditions (U, I, D and ID) in cells harboring the CTCFL transgene. ChIP-seq using FLAG antibody to detect the binding of transgenic CTCFL and CTCF. Binding and expression at *Gal3st1* and *Rapgef1* (**A**) and the *Hoxb* cluster (**B**) is shown. **C, D** Volcano plots showing differentially expressed genes in the wild-type (U) versus CTCFL expressing mESCs in the absence of endogenous CTCF (ID condition) (**C**) and wild type (U) versus CTCF degraded (I condition) cells (**D**). Red and blue points mark the genes with significantly increased or decreased expression respectively (FDR<0.01). The x-axis shows the log2 fold-change in expression and the y-axis the log

10 (False discovery rate) of a gene being differentially expressed. The number of genes that are significantly up or downregulated are indicated in either case. **E** Venn diagrams showing the overlap of differentially expressed genes (DEG) between U versus I condition and U versus D condition (in cells harboring the CTCFL transgene).

Fig S3. CTCFL activates cancer testes antigens (CTA) and components of cancer relevant signaling pathways. A Distribution of peaks bound exclusively by CTCF and/or CTCFL at all binding sites, binding sites in promoters and in promoters of differentially expressed genes (DEG) when CTCFL is expressed (D condition). Average profiles are shown below the corresponding heatmaps. **B-G** IgV tracks showing RNA-seq in all four conditions (U, I, D and ID) in cells harboring the CTCFL transgene. ChIP-seq was performed using a FLAG antibody to detect the binding of transgenic CTCFL and CTCF. Binding of CTCFL and CTCF and transcriptional changes are shown for *Dll1* (**B**) *MageB4, MageE1, MageF1* (**C**) *Adamts1, Adamts2, Adamts15* (**D**) *Tgfb1* and *Stat1* (**E**) *Bhlhe40, Klf10* and *Gadd45b* (**F**) and *Cited1* (**G**).

Fig S4. The impact of CTCFL on 3D chromatin organization. A Hi-C analysis was performed in cells harboring CTCF and CTCFL transgenes under U, I, D and ID conditions in replicates. Statistical report showing the number (in millions) of reads assigned to the different categories following the filtering of reads using HiC bench [73]. Only “ds accepted-intra” and “ds accepted inter” reads were used for further analysis. **B** Snapshot of HiC data from Juicebox showing loss of loops when CTCF is degraded (CTCFL-I) and rescue under conditions of CTCFL-D and CTCF-ID, but not CTCFL-ID. The loop regions from Hiccups and Flag ChIPs corresponding to the respective cell lines are shown along the x-and y-axis.

Fig S5. The role of CTCF and CTCFL zinc fingers and N/C terminal regions in site specific binding. A Flow cytometry confirms the comparable level of mRuby2 expression of transgenic CTCF,

CTCFL, LCL and CLC. **B** IgV tracks of RNA-seq showing expression of the transgenes. At the *Ctcfl* locus, peaks are seen at the exons corresponding to the swapped regions with the chimeric proteins, all exons with *Ctcfl* and no peaks with the *Ctcf* transgene. **C, D** Heatmap showing the clustering of ChIP-seq signals where CTCF, CTCFL, CLC and LCL bind in the presence (D) and absence (ID) of endogenous CTCF. The peak sites corresponding to CTCF only, CTCFL only and CTCF and CTCFL overlapping sites were used for the analysis. These were sorted by site in (**C**) but not in (**D**). **E**. Immunoprecipitation of endogenous GFP tagged CTCF using GFP-Trap and blotting with FLAG antibody demonstrate an interaction between CTCF and transgenic FLAG tagged CTCF, CLC and LCL but not CTCFL. The blot with the GFP antibody shows the pull down of endogenous CTCF in cells expressing the different transgenes.

Fig S6. Impact of fusion proteins on gene expression. A-D IgV tracks showing RNA-seq of indicated conditions in cells harboring CTCF, CTCFL, CLC or LCL transgene under the indicated conditions. ChIP-seq using FLAG antibody was used to detect the binding of transgenes. Binding and expression at *Gadd45g* (**A**), *Igf2* (**B**), *Prss50*, *Steap1* (**C**) and *Egr1* (**D**) is shown.

Fig S7. The impact of fusion proteins on chromatin organization. A Snapshot of Hi-C data from Juicebox corresponding to Chr 8: 63,616,214-69,456,200 8: 63,566,214- 69,406,200 at 10 kb resolution. Cells harboring CTCF, CTCFL, CLC or LCL transgenes were treated with Dox as indicated for 4 days. The corresponding FLAG ChIPs are shown on the x-and y-axis as indicated. **B** The total number (**B**) and mean length (**C**) of TADs called in cells harboring transgenic CTCF, CTCFL, CLC and LCL when treated with D or ID as indicated for 4 days. Untreated (U) as well as CTCF degraded (I) cells harboring CTCF transgenes serve as positive and negative controls.
