# Supplementary material for: Defining the relative and combined contribution of CTCF and CTCFL to genomic regulation
Source: Genome Biol. 2020 May 11;21:108. doi: 10.1186/s13059-020-02024-0 (PMC7212617; doi:10.1186/s13059-020-02024-0)
Supplement: Supplementary file 4 — Table S3. Information regarding experiments performed with the transgenes under different conditions and the figures where the relevant data is shown. [file 13059_2020_2024_MOESM4_ESM.pdf]

Transgene	Condition	Experiments	Figure
CTCF	U	RNA seq	Fig. 5a, 5d<br>Figure S2B, S2C, S6
		HiC	Figure S4A, S7B, S7C
	I	RNA seq	Fig. 5a, 5d<br>Figure S2B, S2C, S6
		HiC	Fig 6c, 6d, 7g-i<br>Figure S4A, S7B, S7C
	D	FLAG ChIPmentation	Fig. 2b, 4c, 7d
		RAD21 ChIPmentation	Fig. 2b, 4c
		HiC	Fig 3<br>Figure S4A, S5B, S5C, S7A
	ID	FLAG ChIPmentation	Fig. 2a-e, 4d-h, 7e<br>Figure S2D, S2E, S3A-E, S3G, S5B, S6, S7B, S7C
		HiC	Fig 3, 6a, 6a-d, 7g-i<br>Figure S4A, S4B, S5B, S5C
	ID +<br>RNase treatment	FLAG ChIPmentation	Fig. 2g
CTCFL	U	RNA seq	Fig. 2a, 2f, 5a, 5d<br>Figure S2A, S2C, S2D, S2E, S3A-E, S6
		HiC	Fig 3<br>Figure S4A, S4B
	I	RNA seq	Fig. 2a<br>Figure S2D, S2E, S3A-E
		HiC	Fig 3<br>Figure S4A, S4B
	D	RNA seq	Fig. 2a, 2f, 5a, 5d<br>Figure S2C, S2D, S2E, S3A-E, S6
		FLAG ChIPmentation	Fig. 2a-e, 4c, 7d<br>Figure S2D, S2E, S3A-E, S3G
		RAD21 ChIPmentation	Fig. 2b, 4c
		HiC	Fig 3<br>Figure S4A, S4B, S5B, S5C, S7A, S7B, S7C
	ID	RNA seq	Fig. 2a<br>Figure S2A, S2D, S2E, S3A-E
		FLAG ChIPmentation	Fig. 2a-e, 4d-h, 7e

			Figure S2D, S2E, S3A-E, S5B, S6
		RAD21 ChIPmentation	Fig. 2b
		HiC	Fig 3, 6a-c<br>Figure S4A, S4B, S5B, S5C, S7B, S7C
	ID +<br>RNase treatment	FLAG ChIPmentation	Fig. 2g
CLC	U	RNA seq	Fig. 5a, 5c, 5f<br>Figure S6
	D	FLAG ChIPmentation	Fig. 4c, 4g, 4h, 7d<br>, S6
		RAD21 ChIPmentation	Fig. 4c
		RNA seq	Fig. 5a, 5c<br>Figure S6
		HiC	Figure S5B, S5C, S7A, S7B, S7C
	ID	FLAG ChIPmentation	Fig. 4d-h, 7e<br>Figure S5B
		RNA seq	Fig. 5d, 5f
		HiC	Fig. 6a-d<br>Figure S5B, S5C, S7B, S7C
LCL	U	RNA seq	Fig. 5a, 5b, 5e<br>Figure S6
	D	FLAG ChIPmentation	Fig. 4c, 4g, 4h<br>Figure S6
		RAD21 ChIPmentation	Fig. 4c
		RNA seq	Fig. 5a, 5b<br>Figure S6
		HiC	Figure S5B, S5C, S7A, S7B, S7C
	ID	FLAG ChIPmentation	Fig. 4d-h
		RNA seq	Fig. 5d, 5e
		HiC	Fig. 6a-c, 6d<br>Figure S5B, S5B, S5C, S7B, S7C
CLL	D	FLAG ChIPmentation	Fig. 7d
	ID	FLAG ChIPmentation	Fig. 7e
		HiC	Fig. 7g-i
LLC	D	FLAG ChIPmentation	Fig. 7d
	ID	FLAG ChIPmentation	Fig. 7e
