# Supplementary material for: Defining the relative and combined contribution of CTCF and CTCFL to genomic regulation
Source: Genome Biol. 2020 May 11;21:108. doi: 10.1186/s13059-020-02024-0 (PMC7212617; doi:10.1186/s13059-020-02024-0)
Supplement: Supplementary file 5 — Review history. [file 13059_2020_2024_MOESM5_ESM.docx]

Review History

**First round of review**

**Reviewer 1**

**Are you able to assess all statistics in the manuscript, including the appropriateness of statistical tests used?**

Yes, and I have assessed the statistics in my report.

**Comments to author:**

The manuscript describes an analysis of the contribution of CTCF and CTCFL to gene expression and 3D organization. Authors use a system to deplete the endogenous CTCF protein while expressing CTCFL tagged with FLAG in order to express and detect different protein combinations. The distribution of the two proteins as well as RAD21 was determined by ChIP-seq, and 3D organization by Hi-C of cells expressing different protein combination. Authors also examine the contribution of different domains of the two proteins to their distribution and to the formation of TADs.

The role of CTCFL in 3D organization and its relationship to CTCF has not been examined systematically. Results presented here are important and represent a significant contribution to the field. The manuscript is appropriate for publication in Genome Biology. The following are suggestions the authors may find helpful in preparing a revised version:

1. Authors should clarify the results presented in Figures 1d and 1e. Please describe the expected molecular weights for the CTCF and CTCFL fusion proteins and include MW markers on the sides of the western blots. If I understand correctly, antibodies to FLAG were used for the western in Figure 1d, and these should only recognize CTCFL. Is the upper band CTCF and the lower one CTCFL? If so, why is CTCF recognized by the FLAG antibody? Does the CTCF antibody also recognize CTCFL? Were two different cell lines, independently expressing CTCF and CTCFL used in these experiments or only one cell line expressing both? Please explain in the manuscript so that the figure is more intuitive. Part of the problem may be when and where the panels are cited in the text.

2. There seems to be a similar issue with Figure 2a. I apologize if this is my failure to understand something. Based on the diagram in Figure 1b, only CTCFL is tagged with FLAG, so the ChIP with FLAG antibody shown in Figure 2a should only show signal for CTCFL, and the CTCFL (ID) and CTCF+CTCFL (D) tracks should be the same, which they are because there should only be signal corresponding the CTCFL. If so, why is one track labeled as "CTCF+CTCFL (D)"? Why is the bottom track labeled CTCF (ID) and what is the signal in this track if the CTCF protein is not tagged with FLAG? This is a recurrent problem throughout the manuscript

3. Figure S2. Although the authors provide the names of the main genes dysregulated by alterations in CTCFL and CTCF in this supplementary figure, readers may benefit from having access to the full data in a supplementary table.

4. Lines 250-253. These statements may be too strong. First, authors define promoters as being 3 kb, which will also include regulatory sequences. Second, the difference is only between 22% and 38%. And third, "predominantly" is only 60%.

5. Figures S7a and b. The information in these figures is difficult to read properly. Instead, authors should provide a supplementary table with all the quality control steps of the Hi-C data, including number of replicates, total reads sequenced, reads mapping uniquely, number of inter- and intra-chromosome contacts, and number of contacts below and above 20 kb. Results from Juicer would be ideal and sufficient.

6. Authors may consider showing the results in Figures 3b and 3c as subtraction heatmaps to more easily appreciate differences between conditions.

7. Figure 3d. It would be interesting to see these changes at a specific site, in addition to the aggregate analysis. If the data is of good quality, authors should be able to show this information in a supplementary figure.

8. Figure 3e. It is difficult to appreciate in the figure the changes authors describe in the text. It appears that all signal, including the diagonal, is lower in the heatmap on the right. Perhaps it would be easier to appreciate the differences by using subtraction heatmaps between the two conditions. In this part of the manuscript, including the figure legend, authors talk at the same time about "TAD structure" and "loop strength", which I find confusing. Are the two the same or different? Please clarify.

9. Figure 4 and lines 358-377. When CTCF and CTCFL colocalize, is there only one binding motif or two? If the former, does this mean that there are two populations of cells used in the ChIP experiments, one with bound CTCF and one with bound CTCFL? If this is the case, how does CTCF facilitate interactions with CLC? Is the idea that CTCF binds, interacts with CLC, it comes off, and then CLC binds? This situation would be different from the clustering experiments cited by the authors in which CTCF proteins bound at distant sites cluster by binding RNA.

10. Figure 6a. This information would be easier to understand if it was shown in subtraction heatmaps. In most Hi-C heatmaps shown in the manuscript, distances and chromosomal locations are very difficult to see. Authors should use a different scale with larger text, as they did in Figure 3e. Also, all heatmaps should show scales for interaction frequency.

Minor comments

1. Lines 68-69. "can stop the movement of cohesin movement on chromatin". Replace for something like "can stop the movement of cohesin on chromatin" or "can stop cohesin movement on chromatin".

2. Authors seem to suggest that when CTCF binds close to promoters, it acts as a "transcription factor". This is not necessarily the case, since its presence at promoters could serve to bring enhancers close to the promoter by forming loops with CTCF sites present at enhancers.

3. Lines 103-104. Reference and discuss recent manuscript by Lobanenkov in PNAS.

4. The last 3 paragraphs of the introduction, lines 114-149, where the authors summarize the results, is too long and should be condensed to describe only the salient findings.

5. Lines 197-199. In the introduction, authors mention that CTCFL is expressed in germ line cells before miosis. Are stra8 and Prss50 expressed in these cells and at this time or in other cells of the testis?

6. Line 207. "We identified highlighted 16,809 CTCF-only….). Please explain what is meant by "highlighted".

7. Lines 211-218. RAD21 does not really bind DNA. It localizes at sites of CTCF because its extrusion is interrupted by CTCF. The wording in this paragraph should reflect this.

8. Lines 233-234. Please describe the co-occurrence of CTCF and CTCFL without using language that may bias the reader. 60% does not suggest "typically" and 55% is not "primarily".

9. In the supplementary Figure legends, Figures S4 and S5 are described as Figure 4 and Figure 5.

10. Line 376. "the N/C domain or CTCF" should be "the N/C domain of CTCF".

11. Line 745. "to a final of 0.125 M" should probably read "to a final concentration of 0.125 M"

**Reviewer 2**

**Are you able to assess all statistics in the manuscript, including the appropriateness of statistical tests used?**

No, I do not feel adequately qualified to assess the statistics.

**Comments to author:**

In this study the authors have comprehensively characterized similarities and differences between the sequence specific DNA binding proteins CTCF, which is ubiquitously expressed, and its testis specific paralog CTCFL, in the context of genome architecture and gene regulation. CTCF is thought to function as a boundary for loop extruding cohesin complexes and - as a consequence of this function - to define the anchors of chromatin loops and the borders of topologically associating domains (TADs). Both CTCF and CTCFL have been implicated in gene regulation, but whether CTCFL also contributes to genome architecture is unknown.

CTCF and CTCFL share a highly conserved zinc finger DNA binding domain but have unique N and C terminal domains. Normally, ubiquitous expression of CTCF is complemented with CTCFL expression in male pre-meiotic germs cells in testis only, however aberrant CTCFL expression has been observed in many cancers. How aberrantly expressed CTCFL affects CTCF's function in genome architecture and gene regulation is also poorly understood.

To answer these questions, the authors used a complementation system which enabled them to analyze cellular functions of ectopically expressed CTCF and CTCFL, expressed either as wildtype proteins or in chimeric forms in which zinc fingers and N- and C-terminal domains of both proteins were fused in different combinations. In these experiments endogenous CTCF could be depleted by auxin induced degradation. The authors used this system to dissect the relative and combined contribution of individual domains of CTCF and CTCFL to their genome-wide binding patterns, effect on cohesin distribution in the genome, regulation of gene expression and genome architecture.

The main observations from these experiments are:

1. CTCFL cannot substitute for CTCF in retaining cohesin at shared sites and cannot restore insulation between TADs and loops that are lost after CTCF depletion.

2. CTCF's N terminal domain (79 aa) is required but not sufficient to rescue insulation between TADs and loops.

3. The zinc finger domains of CTCF and CTCFL but also their N and C terminal domains contribute to binding site specificity.

4. CTCFL has a certain preference for binding to promoters for CTCFL, whereas CTCF is more often found in intergenic and intronic regions.

5. Both proteins have effects on gene expression when bound to promoters but regulate different genes.

6. The authors also claim that CTCF directly binds to cohesin via CTCF's N-terminus, whereas CTCFL is unable to physically interact with cohesin.

This study addresses important questions and provides novel insights that will be relevant for understanding genome architecture, gene regulation and cancer biology, and most of these conclusions are well supported by the experimental data. One exception is the claim that cohesin binds directly to the N-terminus of CTCF, but not to CTCFL. I would therefore recommend publication of this manuscript following appropriate revision.

Specific comments

1. The experimental system is elegant, but it is not clear from the data presented how the levels of AID tagged proteins compare to their untagged counterparts, and likewise how the levels of the ectopically expressed proteins compare to the levels of both untagged and AID-tagged proteins. The first question should be addressed because the AID system is known to be "leaky", i.e. to reduce the levels of tagged proteins even in the absence of auxin. These comparisons would be important for the interpretation of the results.

2. The results from the co-immunoprecipitation experiments are not convincing. For example, in Figure 3f very little Rad21 signal can be detected in the CTCF-IP, and this is right next to the strong input signal, making it difficult to exclude that the weak signal in the IP Is simply spill-over from the input. It would be better to load these samples on the same gel but not next to each other. I have similar concerns about Figure 6D and 7E. In the latter figure it would in addition be important to show that LLC did not co-IP cohesin. The conclusion that these physical interactions explain the CTCF convergence rule is also overstated. It would be more appropriate to say that these interactions are consistent with this rule. Alternatively, if the quality of these co-IP experiments cannot be improved the conclusions would have to be amended. This would also be acceptable and the large body of other interesting data in the manuscript would still justify its publication.

**Authors Response**

**Point-by-point responses to the reviewers’ comments:**

*We would like to thank the reviewers for taking the time to read our manuscript and provide us with helpful and constructive feedback. We have addressed all comments as outlined below.*

*In addition, we have included experiments that we believe will further strengthen the paper. All new experiments and alterations to the manuscript are highlighted in the text in blue.*

*1. We have now included the results of ChIP-seq experiments with CTCF and CTCFL following RNase treatment showing that the RNA interaction is only necessary for CTCF binding and its degradation does not affect CTCFL binding.*

*2. We performed GFP trap experiments where endogenous CTCF was pulled down with the GFP tag and its interaction with transgenic CTCF, CTCFL, CLC and LCL was visualized by blotting using an antibody against the FLAG tag (Fig. S5E). This experiment demonstrates that endogenous CTCF can interact with transgenic CTCF, CLC and LCL but not with CTCFL. Although the results are clear and consistent between experiments the quality of the gel is not optimal even after several tries. We will leave it up to the reviewers to decide whether this new data is good enough to be included or not.*

*3. We performed more detailed analysis of insulation scores using HiCratio [1] instead of the previously used Crane method in our initial submission [2]. Using HiCratio, boundary insulation ratio scores are measured using 40 kb windows across the genome. TAD boundaries are then identified as the local maxima of the insulation scores for each chromosome. We determined the false discovery rate by repeating the same analysis on perturbed matrices. TAD boundaries were reported at 5% false discovery rate. We previously used the Crane algorithm in which Mean Boundary Scores are calculated using a larger window since multiple 40 kb bins fall within one Crane boundary. HiCratio eliminates this step to avoid discrepancy in calculating an accurate insulation score in order to robustly call TAD boundaries. With this more robust analysis we can demonstrate that both CLC and LCL chimeric transgenes can partially rescue the insulation score that is lost upon CTCF depletion. This is consistent with our APE and TAD analysis.*

*4. We now include Hi-C experiments using cells expressing transgenic chimeric CTCFL harboring the CTCF N terminal (CLL) that can partially rescue TADs, loops and insulation scores in the absence of CTCF, highlighting the role played by the CTCF N terminus in chromatin folding. These functions can be explained by CLL’s ability to interact with RAD21.*

*5. Considering the number of transgenes, treatment conditions and experimental methods that we use, we have now included a new Table compiling this information (Table S3). We believe that this will make it easier for the readers to navigate through the different condition*s.

Reviewer reports:

Reviewer #1:

The manuscript describes an analysis of the contribution of CTCF and CTCFL to gene expression and 3D organization. Authors use a system to deplete the endogenous CTCF protein while expressing CTCFL tagged with FLAG in order to express and detect different protein combinations. The distribution of the two proteins as well as RAD21 was determined by ChIP-seq, and 3D organization by Hi-C of cells expressing different protein combination. Authors also examine the contribution of different domains of the two proteins to their distribution and to the formation of TADs.

The role of CTCFL in 3D organization and its relationship to CTCF has not been examined systematically. Results presented here are important and represent a significant contribution to the field. The manuscript is appropriate for publication in Genome Biology. The following are suggestions the authors may find helpful in preparing a revised version:

1. Authors should clarify the results presented in Figures 1d and 1e. Please describe the expected molecular weights for the CTCF and CTCFL fusion proteins and include MW markers on the sides of the western blots. If I understand correctly, antibodies to FLAG were used for the western in Figure 1d, and these should only recognize CTCFL. Is the upper band CTCF and the lower one CTCFL? If so, why is CTCF recognized by the FLAG antibody? Does the CTCF antibody also recognize CTCFL? Were two different cell lines, independently expressing CTCF and CTCFL used in these experiments or only one cell line expressing both? Please explain in the manuscript so that the figure is more intuitive. Part of the problem may be when and where the panels are cited in the text.

*We have now included the molecular weights in the legend for Fig. 1d. “CTCF has a predicted molecular weight of 84 KDa and CTCFL, 74 KDa. However, CTCF is known to migrate as a 130 kDa protein [3]. The chimeric transgenes are expressed with a FLAG tag and mRuby2 which together adds another 29 kDa, so the resulting proteins migrate at 159 and 103 KDa respectively.” The western blot gels of CTCF and CTCFL in Fig. 1d and e are now replaced so as to include the MW markers.*

*There are two different cell lines mentioned in Fig. 1, one expressing transgenic CTCFL and the other expressing control transgenic CTCF. This is explained in the text: “To study the impact of CTCFL expression, we established a rescue system wherein the ESC degron cell line was modified to express either a stable doxycycline-inducible CTCFL or control wild-type CTCF transgene (Fig. 1b,c)”. Both the transgenes have an N terminal FLAG tag. For increased clarity, Fig. 1b has now been modified to show both the CTCFL and CTCF transgenic constructs.*

*In Fig. 1d and e, a Western blot with FLAG antibody was performed on cells expressing transgenic CTCF and CTCFL. The upper and lower bands are CTCF and CTCFL, respectively. The CTCF antibody we used (Active Motif # 61311) recognizes the N terminal portion of CTCF and does not interact with CTCFL.*

2. There seems to be a similar issue with Figure 2a. I apologize if this is my failure to understand something. Based on the diagram in Figure 1b, only CTCFL is tagged with FLAG, so the ChIP with FLAG antibody shown in Figure 2a should only show signal for CTCFL, and the CTCFL (ID) and CTCF+CTCFL (D) tracks should be the same, which they are because there should only be signal corresponding the CTCFL. If so, why is one track labeled as "CTCF+CTCFL (D)"? Why is the bottom track labeled CTCF (ID) and what is the signal in this track if the CTCF protein is not tagged with FLAG? This is a recurrent problem throughout the manuscript

*As mentioned above CTCF and CTCFL are both tagged with FLAG, albeit in different cell lines. Thus CTCFL-ID shows peaks where CTCFL binds in the absence of endogenous CTCF and CTCFL-D shows peaks where CTCFL binds in the presence of endogenous CTCF. The bottom track (CTCF-ID) shows the regions where transgenic CTCF binds in the absence of endogenous CTCF. In all cases, the protein that is FLAG tagged is underlined.*

*This was explained in the legend of Fig. 2a as follows: “FLAG ChIP-seqs to detect CTCFL and CTCF in cells with the respective transgenic knock-ins. The protein whose binding is being assessed is underlined.”*

3. Figure S2. Although the authors provide the names of the main genes dysregulated by alterations in CTCFL and CTCF in this supplementary figure, readers may benefit from having access to the full data in a supplementary table.

*Thank you for the suggestion. We have now included the full list of differentially expressed genes after CTCF degradation (U versus I), CTCF degradation and expression of CTCFL (U versus ID) and CTCFL expression in the presence of CTCF (U versus D) as Table S1.*

4. Lines 250-253. These statements may be too strong. First, authors define promoters as being 3 kb, which will also include regulatory sequences. Second, the difference is only between 22% and 38%. And third, "predominantly" is only 60%.

*This is now edited appropriately as follows: “CTCF only sites have a preference for intronic and intergenic regions, while in contrast, CTCFL-only sites favored promoters (Fig. 2e). Overall, only 22% of CTCF sites were at promoters, versus 38% for CTCFL (Fig. 2e). CTCF binding at promoters co-occurred with CTCFL in 60% of cases, while CTCFL binding at promoters occurred without CTCF in 55% of cases.”*

5. Figures S7a and b. The information in these figures is difficult to read properly. Instead, authors should provide a supplementary table with all the quality control steps of the Hi-C data, including number of replicates, total reads sequenced, reads mapping uniquely, number of inter- and intra-chromosome contacts, and number of contacts below and above 20 kb. Results from Juicer would be ideal and sufficient.

*This is a good suggestion. Accordingly, we have now included Table S2 that gives the HiC-bench filter stats (a statistics summary of automatically generated filtered Hi-C reads) of the two replicate experiments performed in each case. We have removed Figures S4B, S7A and B from the new version.*

6. Authors may consider showing the results in Figures 3b and 3c as subtraction heatmaps to more easily appreciate differences between conditions.

*This is a good suggestion and we now include subtraction heatmaps for the following comparisons (CTCF U - I, CTCFL U - D, CTCF ID – CTCFL ID) in Fig. 3c.*

7. Figure 3d. It would be interesting to see these changes at a specific site, in addition to the aggregate analysis. If the data is of good quality, authors should be abe to show this information in a supplementary figure.

*We agree with the reviewers. The data corresponding to a specific example is now included in Figure S4B.*

8. Figure 3e. It is difficult to appreciate in the figure the changes authors describe in the text. It appears that all signal, including the diagonal, is lower in the heatmap on the right. Perhaps it would be easier to appreciate the differences by using subtraction heatmaps between the two conditions. In this part of the manuscript, including the figure legend, authors talk at the same time about "TAD structure" and "loop strength", which I find confusing. Are the two the same or different? Please clarify.

*We have now included subtraction heatmaps in Fig. 3e. The weaker signal across the panels is explainable as all interactions are affected by CTCFL expression. TADs are the regions of chromatin that exhibit high self-interaction and remain insulated from the surrounding regions. They show up as triangles on Hi-C contact maps whose intensity represents the “TAD strength”. On the other hand, “loops” refer to specific regions that interact with one another. They appear as dots at the apex of TADs, the intensity of which defines “loop strength”. We have now included this information in the legend of Fig. 3.*

9. Figure 4 and lines 358-377. When CTCF and CTCFL colocalize, is there only one binding motif or two? If the former, does this mean that there are two populations of cells used in the ChIP experiments, one with bound CTCF and one with bound CTCFL? If this is the case, how does CTCF facilitate interactions with CLC? Is the idea that CTCF binds, interacts with CLC, it comes off, and then CLC binds? This situation would be different from the clustering experiments cited by the authors in which CTCF proteins bound at distant sites cluster by binding RNA.

*As the ChIP experiments were performed with two populations of cells, one with FLAG tagged CTCF and the other with FLAG tagged CTCFL we are only capturing one motif at a time. This is now made clear in Fig. 1.*

*As mentioned above, we have now performed GFP trap experiments to show interaction of endogenous CTCF with transgenic CTCF, CTCFL, CLC and LCL in the Dox condition where endogenous CTCF is present (Figure S5E). Accordingly, CTCF can interact with CLC and LCL but not CTCFL. The interaction between CTCF and CLC could be both competitive and cooperative. However, we cannot really comment about the sequence of the events.*

10. Figure 6a. This information would be easier to understand if it was shown in subtraction heatmaps. In most Hi-C heatmaps shown in the manuscript, distances and chromosomal locations are very difficult to see. Authors should use a different scale with larger text, as they did in Figure 3e. Also, all heatmaps should show scales for interaction frequency.

*We have now included a new panel, Fig. 6b to show the subtraction heatmaps corresponding to CTCF ID – CTCFL ID, CTCF ID – CLC ID and CTCF ID – LCL ID. We have used a larger font for chromosomal locations and all heatmaps now have the interaction frequency.*

Minor comments

1. Lines 68-69. "can stop the movement of cohesin movement on chromatin". Replace for something like "can stop the movement of cohesin on chromatin" or "can stop cohesin movement on chromatin".

*This is now altered to “can stop the movement of cohesin on chromatin”.*

2. Authors seem to suggest that when CTCF binds close to promoters, it acts as a "transcription factor". This is not necessarily the case, since its presence at promoters could serve to bring enhancers close to the promoter by forming loops with CTCF sites present at enhancers.

*This portion is now corrected as: “This implies that, at sites where CTCF binding overlaps with that of CTCFL at promoters, CTCF and CTCFL may act as transcription factors or alternatively CTCF might bring enhancers closer to promoters by forming loops with CTCF sites present at enhancers.”*

3. Lines 103-104. Reference and discuss recent manuscript by Lobanenkov in PNAS.

*We now include a paragraph in the discussion relating to the findings from the Lobanenkov lab: “ While our manuscript was under revision a paper was published by Pugacheva et al., showing that the N terminal region of CTCF is essential but not sufficient for cohesin retention at CTCF sites [62]. Here the authors examined the binding profiles of cohesin and CTCF but did not demonstrate physical interaction between the two proteins, as we have done here. Their study also demonstrated that CTCFL failed to retain cohesin on chromatin [62]. Although the paper draws some of the same conclusions as ours, the experimental strategy differs in two major ways. First, the cell lines used by Pugacheva et al. expressed an endogenous version of CTCF in which only ZFs 1-8 were functional (ZFs 9-11 mutated), while in our case the auxin inducible degron system allowed us to analyze CTCF and CTCFL chimeric proteins in the presence and absence of endogenous CTCF so we could tease apart their individual effects. Second, their analysis of CTCFL and CTCF-CTCFL chimeric proteins was restricted to the 5,000 sites that either lost or reduced CTCF occupancy when mutant CTCF was expressed, while our study involved a global analysis of CTCF, CTCFL and chimeric proteins.”.*

4. The last 3 paragraphs of the introduction, lines 114-149, where the authors summarize the results, is too long and should be condensed to describe only the salient findings.

*We have condensed this part of the introduction as suggested by the reviewer.*

5. Lines 197-199. In the introduction, authors mention that CTCFL is expressed in germ line cells before miosis. Are stra8 and Prss50 expressed in these cells and at this time or in other cells of the testis?

*STRA8 and PRSS50 are testes specific proteins. STRA8 and CTCFL have an overlapping expression pattern while Prss50 is expressed during spermatogenesis in CTCFL positive cells as well as in subsequent stages of development when CTCFL is no longer expressed [5]. This is now added to the text.*

6. Line 207. "We identified highlighted 16,809 CTCF-only….). Please explain what is meant by "highlighted".

*Thank you for pointing out the mistake. “Highlighted” is now removed.*

7. Lines 211-218. RAD21 does not really bind DNA. It localizes at sites of CTCF because its extrusion is interrupted by CTCF. The wording in this paragraph should reflect this.

*We agree with the reviewer. This is now corrected as: “In agreement with previous studies [5-9], we found that CTCF and cohesin have an overlapping binding profile as CTCF blocks the cohesin-mediated extrusion of DNA (Fig. 2b). RAD21 was localized only at sites where CTCF normally binds i.e. CTCF only and CTCF + CTCFL sites but it failed to localize at CTCFL only sites (Fig. 2b).”*

8. Lines 233-234. Please describe the co-occurrence of CTCF and CTCFL without using language that may bias the reader. 60% does not suggest "typically" and 55% is not "primarily".

*These words are now removed.*

9. In the supplementary Figure legends, Figures S4 and S5 are described as Figure 4 and Figure 5.

*These figure legends are now correctly relabeled.*

10. Line 376. "the N/C domain or CTCF" should be "the N/C domain of CTCF".

*The text is now corrected.*

11. Line 745. "to a final of 0.125 M" should probably read "to a final concentration of 0.125 M"

*The change is now incorporated.*

Reviewer #2: ===

In this study the authors have comprehensively characterized similarities and differences between the sequence specific DNA binding proteins CTCF, which is ubiquitously expressed, and its testis specific paralog CTCFL, in the context of genome architecture and gene regulation. CTCF is thought to function as a boundary for loop extruding cohesin complexes and - as a consequence of this function - to define the anchors of chromatin loops and the borders of topologically associating domains (TADs). Both CTCF and CTCFL have been implicated in gene regulation, but whether CTCFL also contributes to genome architecture is unknown.

CTCF and CTCFL share a highly conserved zinc finger DNA binding domain but have unique N and C terminal domains. Normally, ubiquitous expression of CTCF is complemented with CTCFL expression in male pre-meiotic germs cells in testis only, however aberrant CTCFL expression has been observed in many cancers. How aberrantly expressed CTCFL affects CTCF's function in genome architecture and gene regulation is also poorly understood.

To answer these questions, the authors used a complementation system which enabled them to analyze cellular functions of ectopically expressed CTCF and CTCFL, expressed either as wildtype proteins or in chimeric forms in which zinc fingers and N- and C-terminal domains of both proteins were fused in different combinations. In these experiments endogenous CTCF could be depleted by auxin induced degradation. The authors used this system to dissect the relative and combined contribution of individual domains of CTCF and CTCFL to their genome-wide binding patterns, effect on cohesin distribution in the genome, regulation of gene expression and genome architecture.

The main observations from these experiments are:

1. CTCFL cannot substitute for CTCF in retaining cohesin at shared sites and cannot restore insulation between TADs and loops that are lost after CTCF depletion.

2. CTCF's N terminal domain (79 aa) is required but not sufficient to rescue insulation between TADs and loops.

3. The zinc finger domains of CTCF and CTCFL but also their N and C terminal domains contribute to binding site specificity.

4. CTCFL has a certain preference for binding to promoters for CTCFL, whereas CTCF is more often found in intergenic and intronic regions.

5. Both proteins have effects on gene expression when bound to promoters but regulate different genes.

6. The authors also claim that CTCF directly binds to cohesin via CTCF's N-terminus, whereas CTCFL is unable to physically interact with cohesin.

This study addresses important questions and provides novel insights that will be relevant for understanding genome architecture, gene regulation and cancer biology, and most of these conclusions are well supported by the experimental data. One exception is the claim that cohesin binds directly to the N-terminus of CTCF, but not to CTCFL. I would therefore recommend publication of this manuscript following appropriate revision.

Specific comments

1. The experimental system is elegant, but it is not clear from the data presented how the levels of AID tagged proteins compare to their untagged counterparts, and likewise how the levels of the ectopically expressed proteins compare to the levels of both untagged and AID-tagged proteins. The first question should be addressed because the AID system is known to be "leaky", i.e. to reduce the levels of tagged proteins even in the absence of auxin. These comparisons would be important for the interpretation of the results.

*Since both the alleles of endogenous CTCF are AID tagged there is no untagged endogenous counterpart for CTCF. The level of ectopically tagged CTCF is shown in Fig. 1d. The transgenes are always expressed at lower levels than that of the endogenous CTCF. The comparison between endogenous and transgenic CTCF is shown in Fig. 1e. Given the differences in expression between endogenous and transgenic CTCF, all comparisons in the paper are between transgenic (and not endogenous) CTCF and other transgenes whose levels are comparable.*

2. The results from the co-immunoprecipitation experiments are not convincing. For example, in Figure 3f very little Rad21 signal can be detected in the CTCF-IP, and this is right next to the strong input signal, making it difficult to exclude that the weak signal in the IP Is simply spill-over from the input. It would be better to load these samples on the same gel but not next to each other. I have similar concerns about Figure 6D and 7E. In the latter figure it would in addition be important to show that LLC did not co-IP cohesin. The conclusion that these physical interactions explain the CTCF convergence rule is also overstated. It would be more appropriate to say that these interactions are consistent with this rule. Alternatively, if the quality of these co-IP experiments cannot be improved the conclusions would have to be amended. This would also be acceptable and the large body of other interesting data in the manuscript would still justify its publication.

*We have now repeated the co-immunoprecipitation experiments corresponding to Figs. 3f, 6d and 7e loading the gels as suggested by the reviewers. We now also include the IP showing that LLC does not co-IP with RAD 21 (Fig. 7f).*

*We have now altered the statement as follows: These interactions are consistent with convergence of CTCF binding being important for loop formation.*

*1. Gong Y, Lazaris C, Sakellaropoulos T, Lozano A, Kambadur P, Ntziachristos P, Aifantis I, Tsirigos A: Stratification of TAD boundaries reveals preferential insulation of super-enhancers by strong boundaries. Nature communications 2018, 9:1-12.*

*2. Crane E, Bian Q, McCord RP, Lajoie BR, Wheeler BS, Ralston EJ, Uzawa S, Dekker J, Meyer BJ: Condensin-driven remodelling of X chromosome topology during dosage compensation. Nature 2015, 523:240.*

*3. Klenova EM, Nicolas RH, Sally U, Carne AF, Lee RE, Lobanenkov VV, Goodwin GH: Molecular weight abnormalities of the CTCF transcription factor: CTCF migrates aberrantly in SDS-PAGE and the size of the expressed protein is affected by the UTRs and sequences within the coding region of the CTCF gene. Nucleic acids research 1997, 25:466-473.*

*4. Pugacheva EM, Kubo N, Loukinov D, Tajmul M, Kang S, Kovalchuk AL, Strunnikov AV, Zentner GE, Ren B, Lobanenkov VV: CTCF mediates chromatin looping via N-terminal domain-dependent cohesin retention. Proceedings of the National Academy of Sciences 2020.*

*5. Sleutels F, Soochit W, Bartkuhn M, Heath H, Dienstbach S, Bergmaier P, Franke V, Rosa-Garrido M, van de Nobelen S, Caesar L: The male germ cell gene regulator CTCFL is functionally different from CTCF and binds CTCF-like consensus sites in a nucleosome composition-dependent manner. Epigenetics & chromatin 2012, 5:8.*

*6. Parelho V, Hadjur S, Spivakov M, Leleu M, Sauer S, Gregson HC, Jarmuz A, Canzonetta C, Webster Z, Nesterova T: Cohesins functionally associate with CTCF on mammalian chromosome arms. Cell 2008, 132:422-433.*

*7. Pugacheva EM, Rivero-Hinojosa S, Espinoza CA, Méndez-Catalá CF, Kang S, Suzuki T, Kosaka-Suzuki N, Robinson S, Nagarajan V, Ye Z: Comparative analyses of CTCF and BORIS occupancies uncover two distinct classes of CTCF binding genomic regions. Genome biology 2015, 16:161.*

*8. Rubio ED, Reiss DJ, Welcsh PL, Disteche CM, Filippova GN, Baliga NS, Aebersold R, Ranish JA, Krumm A: CTCF physically links cohesin to chromatin. Proceedings of the National Academy of Sciences 2008, 105:8309-8314.*

*9. Wendt KS, Peters J-M: How cohesin and CTCF cooperate in regulating gene expression. Chromosome research 2009, 17:201-214.*

**Second round of review**

**Reviewer 1**

The authors have done an excellent job at addressing all the issues raised by the reviewers. I think the manuscript represents an important contribution to the field and it is appropriate for publication in Genome Biology
